# Nephrolithiasis and renal failure among patients exposed to atazanavir, other PIs and PI-free regimens

**DOI:** 10.7448/IAS.17.4.19560

**Published:** 2014-11-02

**Authors:** Ella Nkhoma, Monica Kumar, Patricia Hines, Vidya Moorthy, Isabelle Klauck, Angelina Villasis Keever

**Affiliations:** 1 Bristol-Myers Squibb, Global Pharmacovigilance and Epidemiology, Wallingford, CT, USA; 2 Bristol-Myers Squibb, Global Pharmacovigilance and Epidemiology, Hopewell, NJ, USA; 3 Bristol-Myers Squibb, Center for Observational Research and Data Science, Hopewell, NJ, USA; 4 Mu Sigma, N/A, Northbrook, IL, USA; 5 Bristol-Myers Squibb, European Medical, Rueil-Malmaison, France; 6 Bristol-Myers Squibb, US Medical, Plainsboro, NJ, USA

## Abstract

**Introduction:**

Recent single-site studies and case reports have linked atazanavir (ATV) with the occurrence of nephrolithiasis. The purpose of this study was to estimate and compare the incidence rate of nephrolithiasis and to characterize the occurrence of subsequent renal failure among patients on ATV, other protease inhibitors (PIs) and PI-free regimens using real world data.

**Materials and Methods:**

This was a retrospective cohort analysis using claims data from a US commercial and a US public health insurance database (Medicaid) spanning 2003–2011 and 2006–2011, respectively. We identified adult HIV patients who were prescribed ATV, other PIs or PI-free regimens with at least 6 months of continuous enrolment prior to the index claim. Nephrolithiasis was defined as an inpatient or outpatient ICD-9 diagnosis code for nephrolithiasis or an associated condition, plus an imaging/corrective procedure code. Renal failure was also identified using diagnosis codes among patients experiencing nephrolithiasis. Hazard ratios were estimated using propensity score (PS) adjusted Cox regression, crude and adjusted for demographics, baseline comorbidities and comedications.

**Results:**

A total of 14,477 patients (ATV: 4,150; other PIs: 4,153; PI-free: 6,174) were identified in the commercial database: 83% male and 20% age ≥50 years. In the Medicaid database, 9,104 patients (ATV: 3,460; other PIs: 3,117; PI-free: 2,527) were identified: 53% male and 25% age ≥50 years. There were significant baseline differences in demographics, comorbidities and concomitant medications among the three cohorts. In adjusted analyses, ATV use was not significantly associated with nephrolithiasis when compared to other PIs. When ATV was compared to PI-free regimens, a positive association was observed in the commercial insurance but not the Medicaid database. In both databases, previous history of nephrolithiasis was the strongest predictor of nephrolithiasis in the ATV/PI-free regimens contrast, but not the ATV/other PIs contrast. For the renal failure outcomes, there were insufficient cases across all cohorts to conduct crude or adjusted analyses (see Table 1).

**Conclusions:**

In this analysis of two large real world databases, we did not find evidence of an increased risk of nephrolithiasis among patients on ATV compared to other PIs. However, when ATV was compared to PI-free regimens, the results differed across the two databases, requiring further study. Additionally, renal failure following nephrolithiasis was infrequent and not significantly different across the three cohorts.

**Table 1:** Incidence rates and hazard ratios of nephrolithiasis and cases of renal failure in patients on atazanavir, other PIs and PI-free regimens

Cohort	Nephrolithiasis cases/person-years (py)	Nephrolithiasis incidence rate per 1,000 py (95% CI)	ATV vs other PIs adjusted HR (95% CI)	ATV vs PI-free adjusted HR (95% CI)	Acute renal failure cases	Chronic renal failure cases
Commercial						
ATV	61/4,865	12.5 (9.4, 15.7)	1.26 (0.85, 1.87)	1.65 (1.15, 2.37)	1	3
Other PIs	44/4,315	10.2 (7.2, 13.2)	Reference		3	0
PI-free	67/8,662	7.7 (5.9, 9.6)		Reference	2	1
Medicaid						
ATV	37/3,460	17.4 (12.2, 23.9)	0.93 (0.58, 1.50)	1.31 (0.74, 2.39)	5	3
Other PIs	34/3,117	20.4 (14.1, 28.5)	Reference		3	0
PI-free	27/2,527	15.1 (10.0, 22.0)		Reference	2	1

Abbreviations: ATV, atazanavir, CI, confidence interval, HR, hazard ratio, PI, protease inhibitor, py, person-years. All comparative analyses were conducted using propensity score stratification adjustment for baseline patient characteristics.

